# Proteomic Profiling of Hematopoietic Stem/Progenitor Cells after a Whole Body Exposure of CBA/CaJ Mice to Titanium (^48^Ti) Ions

**DOI:** 10.3390/proteomes3030132

**Published:** 2015-07-21

**Authors:** Kanokporn Noy Rithidech, Montree Tungjai, Witawat Jangiam, Louise Honikel, Chris Gordon, Xianyin Lai, Frank Witzmann

**Affiliations:** 1 Department of Pathology, Stony Brook University, Stony Brook, NY 11794, USA; E-Mails: mtungjai@gmail.com (M.T.); witawat.jangiam@stonybrookmedicine.edu (W.J.); lmhonikel@hotmail.com (L.H.); chris.gordon@stonybrookmedicine.edu (C.G.); 2 Department of Radiologic Technology, Faculty of Associated Medical Sciences, Center of Excellence for Molecular Imaging, Chiang Mai University, Chiang Mai 50200, Thailand; 3 Department of Chemical Engineering, Burapha University, Chonburi 20131, Thailand; 4 Department of Biochemistry and Molecular Biology, Indiana University School of Medicine, 635 Barnhill Drive, Room 0044, Indianapolis, IN 46202, USA; E-Mail: xlai@iu.edu; 5 Department of Cellular & Integrative Physiology, Indiana University School of Medicine, 635 Barnhill Drive, Room 362A, Indianapolis, IN 46202, USA; E-Mail: fwitzmann@iupui.edu

**Keywords:** space radiation, hematopoietic stem/progenitor cells, mice, proteomics

## Abstract

Myeloid leukemia (ML) is one of the major health concerns from exposure to radiation. However, the risk assessment for developing ML after exposure to space radiation remains uncertain. To reduce the uncertainty in risk prediction for ML, a much increased understanding of space radiation-induced changes in the target cells, *i.e.*, hematopoietic stem/progenitor cells (HSPCs), is critically important. We used the label-free quantitative mass spectrometry (LFQMS) proteomic approach to determine the expression of protein in HSPC-derived myeloid colonies obtained at an early time-point (one week) and a late time-point (six months) after an acute whole body exposure of CBA/CaJ mice to a total dose of 0, 0.1, 0.25, or 0.5 Gy of heavy-ion titanium (^48^Ti ions), which are the important component of radiation found in the space environment. Mice exposed to 0 Gy of ^48^Ti ions served as non-irradiated sham controls. There were five mice per treatment groups at each harvest time. The Trans-Proteomic Pipeline (TPP) was used to assign a probability of a particular protein being in the sample. A proof-of-concept based Ingenuity Pathway Analysis (IPA) was used to characterize the functions, pathways, and networks of the identified proteins. Alterations of expression levels of proteins detected in samples collected at one week (wk) post-irradiation reflects acute effects of exposure to ^48^Ti ions, while those detected in samples collected at six months (mos) post-irradiation represent protein expression profiles involved in the induction of late-occurring damage (normally referred to as genomic instability). Our results obtained by using the IPA analyses indicate a wide array of signaling pathways involved in response to 1 GeV/n ^48^Ti ions at both harvest times. Our data also demonstrate that the patterns of protein expression profiles are dose and time dependent. The majority of proteins with altered expression levels are involved in cell cycle control, cellular growth and proliferation, cell death and survival, cell-to-cell signaling and interaction. The IPA analyses indicate several important processes involved in responses to exposure to ^48^Ti ions. These include the proteosme/ubiquination, protein synthesis, post-translation modification, and lipid metabolism. The IPA analyses also indicate that exposure to 1 GeV/n ^48^Ti ions affects the development and function of hematological system, immune cell trafficking, including the cytoskeleton. Further, the IPA analyses strongly demonstrate that the NF-κB and MAPKs (ERKs, JNKs, and p38MAPK) pathways play an essential role in signal transduction after exposure to 1 GeV/n ^48^Ti ions. At an early time-point (1 week), the top networks identified by the IPA analyses are related to metabolic disease, lipid metabolism, small molecule biochemistry, and development disorder. In contrast, the top networks identified in samples collected at a late time-point (6 mos post-irradiation) by the IPA analyses are related to cancer, hematological disorders, and immunological diseases. In summary, the proteomic findings from our study provide a foundation to uncover compounds potentially be highly effective in radiation countermeasures.

## 1. Introduction

Space environment consists of various types of radiation that are different from those found in the earth’s atmosphere. These include particles with high mass and high energy (HZE) particles such as titanium (^48^Ti) ions. It has been estimated that a manned mission to Mars may take longer than 500 days. During a two-year journey to Mars, every cell in the body may be hit by a HZE particle approximately once a month [1]. Hence, exposure to radiation in space may create potential health risks to astronauts for late occurring diseases such as cancer [2], in particular radiation-induced myeloid leukemia (rML) because it is the type of cancer observed in the atomic bomb survivors at the early time post-exposure and at the lowest doses [3]. However, the predictions of risks for developing ML after exposure to space radiation are inadequate. To increase the reliability of predicting risk for ML, We initiated a study series to evaluate the biological effects of ^48^Ti ions (1 GeV/n, LET = 107 keV/μm) on HSPCs of exposed CBA/CaJ mice. We focus on the HSPC-derived myeloid progenitors since it is believed that they are the target cell for radiation-induced ML (rML) [4,5]. We examined protein expression-profiles in HSPC-derived myeloid colonies (the best population of cells for studying *in vivo* biological effects of radiation on the myeloid lineage of the hematopoietic stem cells) obtained at an early time-point (one week) and a late time-point (six months) after exposure of CBA/CaJ mice to various doses of 1 GeV/n ^48^Ti ions. The use of proteomics in radiation biology is in its infancy, but it offers considerable promise [6]. This is because proteomic analysis delivers information on the molecular and cellular mechanisms that control physiology and pathophysiology of the cell or tissue. The CBA/CaJ mouse was selected as an animal model in this study because existing data indicate that it is an appropriate mouse strain for studying rML [7,8,9]. ^48^Ti ions were selected for study because they are one of the important heavy ions found in the space environment.

Despite the high linear energy transfer (LET) value, very little is known about the *in vivo* biological effects of 1 GeV/n ^48^Ti ions. It was found that exposure of male Sprague-Dawley rats to 0.5 Gy of 1.1 GeV/n ^48^Ti ions disrupted neurobehavioral functions [10]. Further, decreased levels of proteins involved in mitochondrial fatty acid metabolism were found in liver tissues of these exposed rats collected at 20 months [11]. Recently, we [12] found that 1 GeV/n ^48^Ti ions (delivered at 1 cGy/min) induced chronic inflammation (determined by persistently high levels of activated NF-κB and NF-κB related pro-inflammatory cytokines), chronic oxidative stress, and a reduction in the levels of 5-hydroxymethyl-cytosise in the liver of CBA/CaJ mice collected at various times (up to six months) post-irradiation. Of note, these CBA/CaJ mice were the animals that we obtained HSPC-derived myeloid colonies for proteomic analyses being presented in this report.

Using two-dimensional electrophoresis (2-DE) in combination with mass spectrometry, several proteins involved in antioxidant activity, metabolism, signal transduction, and protein post-translational processes have been detected in intestinal epithelial cells isolated from BALB/cJ mice at 3 and 72 h after exposure to a single dose of 9.0 Gy ^137^Cs γ-rays [13]. We found blood-plasma proteins whose expression levels are significantly altered in CBA/CaJ mice exposed to 3 Gy of ^137^Cs γ-rays (a dose known to induce a 25% lifetime incidence of ML in this strain of mouse [14]. The majority of these proteins are involved in inflammatory responses. Our data suggested that alterations in expression-levels of specific proteins in plasma may be indicative of radiation exposure. Our results also provided the important step in an eventual establishment of blood-based biomarkers of radiation-exposure *in vivo*. However, these data do not provide information on radiation-induced changes in the proteome of HSPC-derived myeloid colonies, the target for radiation-induced ML. This study is the first to investigate the alterations in proteomic profiles of HSPC-derived myeloid colonies after exposure of mice to ^48^Ti ions.

Despite the effects of radiation on hematopoietic tissues have been extensively investigated, very little is known about the HSPC proteome after irradiation. In the past, the proteomic-based approaches have been used to search for potential biomarkers of exposure and effects of radiation in the total population of bone marrow (BM) cells, which contains a mixed population (myeloid and lymphoid lineages), both immature and mature cells [15,16]. In one study [15], five groups of proteins were detected in supernatant prepared from BM cells. These are antioxidants (e.g., peroxiredoxin2, superoxide dismutase), carriers (e.g., α-fetoprotein, apolipoprotein A-1, ferritin, haptoglobin (precursor), cytosolic proteins (e.g., serum albumin, transthyretin precursor, carbonic anhydrase, and creatine kinase M chain M), including proteases and protease inhiborts (e.g., α-1-antitrypsin 1-3 and neutrophil collagenase). Among these, serum ablbumin, apolipoprotein A-1, ferritin, haptoglobin and α-1 antitrypsin) were altered in BM cells collected at 24 h from CBA/Ca or C57BL/6 mice exposed to 4 Gy of ^137^Cs γ-rays (delivered at 0.44 Gy/min), in relation to their corresponding non-irradiated sham controls [15]. The authors also found differentially plasma protein expression between these two strains of mouse. The expression levels of α-fetoprotein, neutrophil collagenase precursor and serotransferrin precursor were increased, while peroxiredoxin 2 was decreased in C57BL/6 mice. In contrast, the expression level of cytosolic proteins (creatine kinase M chain) was decreased in CBA/Ca mice. These findings may reflect the known differential sensitivity to radiation of BM cells from these two strains of mouse. These include, BM cells of the CBA/Ca mouse are much more sensitive to radiation-induced chromosome aberrations [17] but less sensitive to radiation-induced apoptosis than those of the C57BL/6 mouse [18,19]; and the CBA/Ca, not the C57BL/6, mouse develops ML after exposure to radiation [17]. Subsequently, we found increased levels of α-1-antitrypsin and apolipoprotein A-1 in blood plasma collected at two or seven days after exposure of the CBA/CaJ to a single dose of 137Cs γ-rays [14]. Further, the level of apolipoprotein A-1 was also increased in blood plasma collected from CBA/CaJ mice developed rML after exposure to radiation [20]. Recently, the high level of carbonylation was detected in specific proteins isolated from BM cells collected at 24 h after exposure of the C57BL/6 mice exposed to a high dose (7.5 Gy) of ^60^Co γ-rays [16]. These proteins include chitinase-like protein 3 (a glycoprotein glycoprotein linked to inflammatory responses) and carbonic anhydrase 2 (a regulator of acid-base homeostasis). To date, the role of carbonylation in these two BM proteins in carcinogenesis is unknown.

Evidently, the results from these BM proteomic studies offer information for acute or early responses of the total BM population only since BM cells were collected at 24 h after irradiation. Further, only high doses of low LET radiation, *i.e.*, 4 [15] or 7.5 [16] Gy of γ-rays, were used. These high doses of radiation are irrelevant to the induction of rML in the mouse model. It is known among the mouse models for rML (*i.e.*, CBA/Ca, CBA/H, CBA/Cne, C_3_H/He, RFM and SJL mice) that a single dose of 2 Gy of X-rays or 3 Gy of ^137^Cs γ-rays induces a 20%–25% lifetime incidence of rML [7,21,22,23,24,25]. The incidence of rML is reduced when higher doses of X- or γ-rays were used, presumably due to cell killing effects. Evidently, the results from these BM proteomic studies do not provide information on HSPC proteome after exposure to radiation. Currently, neither the effect of low LET radiation (at doses below 4 Gy) nor the effect of low doses of ^48^Ti ions on HSPC proteome is available.

Recently, we used a unique label-free quantitative mass spectrometry (LFQMS) platform (the technique developed in our laboratory [26]) to investigate the effects of 300 MeV/n ^28^Si ions (one type of heavy ions found in the space environment) on the proteome of HSPC-derived myeloid colonies obtained at six months after exposure of CBA/CaJ [27]. This is the first study to use a proteomic-based approach to investigate the effects of heavy ions on the proteome of HSPC-derived myeloid colonies. The total doses of 300 MeV/n ^28^Si ions were 0, 0.1, 0.25, or 0.5 Gy (using a fractionated schedule, two exposures, 15 days apart that totaled each selected dose). We identified 1344 unique, non-redundant proteins with ≥90% confidence from 3254 peptides, quantified and the abundance of these proteins was compared statistically. Among the 1344 proteins, levels of expression of 198 proteins were found to be statistically significant in HSPC colonies obtained from treated groups, in relation to those found in non-irradiated sham controls. We characterized the functions and pathways of these 198 identified proteins using the Ingenuity Pathway Analysis (IPA) [28]. The IPA analysis showed that the majority of these proteins are cancer related (*p =* 6.38 × 10^−10^*)*. Biochemical analyses of molecular and cellular functions of these proteins revealed association with perturbation in cell survival, free radical scavenging, cell cycle, DNA repair, cellular assembly, hematological system development, and inflammatory responses. The results also indicated that these proteins are linked to two major molecular networks that are linked to cancer and inflammatory responses: The nuclear factor-kappa B and the protein phasphatase 2 A networks. In this study, we also used the LFQMS proteomic platform to determine the responses of HSPC-derived myeloid colonies obtained at one week and 6 months after exposure of CBA/CaJ mice to various doses of 1 GeV/n ^48^Ti ions at the proteomic level. Of note, the HSPC-derived myeloid colonies were obtained from the same mice used for studying early and late effects of 1 GeV/n ^48^Ti ions on the liver, in which chronic inflammation, oxidative stress, and aberrant patterns of global DNA were detected [12]. 

## 2. Experimental Section

### 2.1. Animals

Details of CBA/CaJ mice used in this study were presented in our previous study investigating the early and late effects of 1 GeV/n ^48^Ti ions on the liver of exposed CBA/CaJ mice [12]. Briefly, a total of 40 male CBA/CaJ mice were purchased from the Jackson Laboratory (Bar Harbor, ME, USA) and were delivered directly to Brookhaven National Laboratory (BNL), Upton, NY, USA where ^48^Ti-irradiation took place. There was a two-week acclimatization period prior to irradiation (at 10–12 weeks of age, with an average body weight of 25 g). Mice were housed in a facility approved by the Association for Assessment and Accreditation of Laboratory Animal Care (AAALAC) at BNL. The animal rooms were maintained with the light cycle of 12 h light/12 h dark, 21 ± 2 °C, with 10–15 hourly cycles of fresh air and a relative humidity of 50% ± 10%. The protocols for animal housing and care, including experimental design of the study, were approved by both the BNL (Upton, NY, USA) and the Stony Brook University (SBU) Institutional Animal Care and Use Committee (Stony Brook, NY, USA).

### 2.2. Irradiation of Mice

Irradiation of mice to 1 GeV/n ^48^Ti ions (LET = 107 keV/μm) was done at the National Aeronautics and Space Administration (NASA), Research Laboratory (NSRL) located at BNL, Upton, NY, USA. The procedure of exposure was presented previously [12]. In brief, mice were exposed whole body to the average total-body doses of 0.1, 0.25, or 0.5 Gy, delivered at the dose rate of 1 cGy/min by a 20 cm × 20 cm beam. Mice (age-matched) exposed to 0 Gy served as sham controls. The selected dose and dose rate of 1GeV/n ^48^Ti ions are comparable to what the astronauts encounter in the space environment. Mice were transported back to SBU in a climate-controlled vehicle within 48 h post-exposure. The animal facility of SBU, where sample collections were performed, is also approved by AAALAC, with the same light cycle (12 h light/12 h dark), temperature (21 ± 2 °C), 10–15 hourly cycles of fresh air, and relative humidity (50% ± 10%) as those of BNL.

### 2.3. Collection of BM Cells for Obtaining Hematopoietic Stem/Progenitor Cells Using the Colony-Forming-Unit Assay (CFU-A)

**Figure 1:**
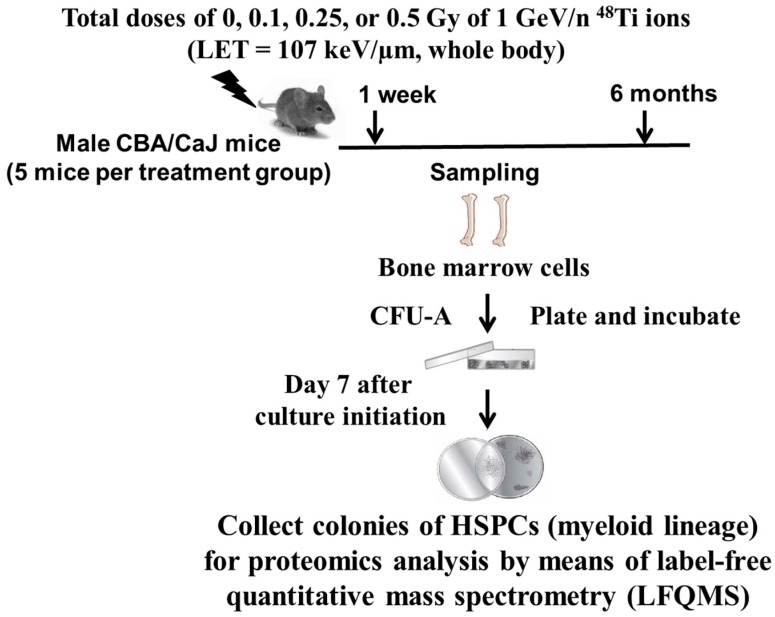
A diagram of the experimental design

This is a serial sacrifice study. There were two harvest times following the exposure to 1 GeV/n ^48^Ti ions, *i.e.*, 1 week, and 6 months. Mice included in the sham-control group were age-matched to exposed mice. Therefore, the age of mice in each treatment group would be similar at each sacrifice time. At each harvest time, for each treatment group, we collected BM cells from each mouse (five mice per dose of ^48^Ti ions). The first day after irradiation was designated as day 1 after exposure. There were three harvest times following the exposure to 1 GeV/n ^48^Ti ions, *i.e.*, 1 week, 1 month, and 6 months. Mice included in the sham-control group were age-matched to exposed mice. Therefore, the age of mice in each treatment group would be similar at each sacrifice time. Sample collected at 1 week post-exposure (for the detection of acute effects) and 6 mos post-exposure (at which time radiation-induced genomic instability has been observed, determined by the occurrence of late-occurring chromosome aberrations [29,30]. We flushed both femurs and tibiae with 10 mL of Iscove’s Modified Dulbecco’s Medium (IMDM) (Invitrogen, Carlsbad, CA, USA). Thereafter, BM cells were seeded on the semi-solid methylcellulose-based medium MethoCult^®^ 3434 purchased from StemCell Technologies (Vancouver, BC, Canada). As suggested by the manufacturer, a total of 2 × 10^4^ BM cells were grown in a 35 mm dish (containing 1.1 mL of MethoCult^®^ 3434 medium). This medium contains several growth factors critically important to support the optimum growth of cells with myeloid lineages: Granulocyte-macrophage progenitors (CFU-GM, CFU-M, CFU-G), multi-potential granulocyte, erythroid, macrophage, megakaryocyte progenitors (CFU-GEMM), including erythroid progenitors (CFU-E, BFU-E). [31]. As shown in Figure 1, at Day 7 after culture initiation, HSPC-derived myeloid colonies were collected for proteomic profiling. Of note, we also scored colonies according to morphology using an inverted light microscope prior to harvesting for analyses.

### 2.4. Proteomics Analyses

#### 2.4.1. Materials

Triethylphosphine, iodoethanol, and ammonium bicarbonate (NH_4_HCO_3_) were purchased from Sigma-Aldrich (St. Louis, MO, USA). LC-MS grade 0.1% formic acid in acetonitrile (ACN) and 0.1% formic acid in water (H_2_O) were purchased from Burdick & Jackson (Muskegon, MI, USA). Modified sequencing grade porcine trypsin was obtained from Princeton Separations (Freehold, NJ, USA).

#### 2.4.2. Protein Extraction

Protein extraction was performed in Rithidech’s Lab. Protein concentration was determined by the Bradford Protein Assay using Bio-Rad protein assay dye reagent concentrate [1]. 

#### 2.4.3. Protein Reduction, Alkylation, and Digestion for LC-MS/MS

Protein reduction, alkylation, and digestion were carried out using a conventional method previously published by Lai *et al.* [32]. Briefly, a 100 μg aliquot of protein sample was placed in a 2 mL tube. The volume of the sample was adjusted to 200 μL. Two hundred microliters of the reduction/alkylation cocktail consisting of 0.5% of triethylphosphine and 2% of iodoethanol was added to the protein solution. The sample was incubated at 35 °C for 60 min, dried by SpeedVac (Jouan, Winchester, VA, USA), and reconstituted with 100 μL of 100 mM NH_4_HCO_3_ at pH 8.0. A 150 μL aliquot of a 20 μg/mL trypsin solution was added to the sample and incubated at 35 °C for 3 h, after which another 150 μL of trypsin was added, and the solution incubated at 35 °C for 3 h

#### 2.4.4. LC-MS/MS

The digested samples were analyzed using a Thermo-Finnigan linear ion-trap (LTQ) mass spectrometer coupled with a Surveyor autosampler and MS HPLC system (Thermo-Finnigan, Waltham, MA, USA). Tryptic peptides were injected onto a C18 reversed phase column (TSKgel ODS-100V, 3 μm, 1.0 mm × 150 mm (Tosoh Bioscience LLC, King of Prussia, PA, USA) at a flow rate of 50 μL/min. The mobile phases A, B, and C were 0.1% formic acid in water, 50% ACN with 0.1% formic acid in water, and 80% ACN with 0.1% formic acid in water, respectively. The gradient elution profile was as follows: 10% B (90% A) for 7 min, 10%–67.1% B (90%–32.9% A) for 163 min, 67.1%–100% B (32.9%–0% A) for 10 min, and 100%–50% B (0%–50% C) for 10 min. The data were collected in the “Data dependent MS/MS” mode with the ESI interface using normalized collision energy of 35%. Dynamic exclusion settings were set to repeat count 1, repeat duration 30 s, exclusion duration 120 s, and exclusion mass width 0.60 *m*/*z* (low) and 1.60 *m*/*z* (high).

#### 2.4.5. Peptide and Protein Identification and Quantification

The acquired data were searched against the UniProt protein sequence database of mouse (released on 11 July 2012) using SEQUEST (version. 28 rev. 12, Thermo-Finnigan, Waltham, MA, USA) algorithms in Bioworks (version 3.3, Thermo-Finnigan). General parameters were set to: Mass type set as “monoisotopic precursor and fragments”, enzyme set as “trypsin(KR)”, enzyme limits set as “fully enzymatic—Cleaves at both ends”, missed cleavage sites set at 2, peptide tolerance 2.0 amu, fragment ion tolerance 1.0 amu, fixed modification set as +44 Da on Cysteine, and no variable modifications used. The searched peptides and proteins were validated by PeptideProphet [33] and ProteinProphet [34] in the Trans-Proteomic Pipeline (TPP, version 3.3.0, Seattle Proteome Center, Seattle, WA, USA). Only proteins with probability ≥0.9000 and peptides with probability ≥0.8000 were reported. Protein quantification was performed using IdentiQuantXL™ software as described [26]. One-way ANOVA (with a statistical significance at *p* < 0.01)) and Pairwise Multiple Comparison (Holm-Sidak method, with a statistical significance at *p* < 0.05) are applied to determine the significance of differences.

## 3. Results and Discussion

### 3.1. Effects of 1 GeV/n ^48^Ti Ions on HSPC-Derived Myeloid Colonies Collected at One Week Post-irradiation

For all exposed groups, a total of 1988 unique and non-redundant proteins were identified with ≥90% confidence (Supplementary data A). Among the 1988 identified, a total of 1107 proteins were differentially expressed based on the one-way analysis of variance (ANOVA, *p* < 0.01). For the multiple comparisons among groups (the Holm-Sidak method, with significant differences at *p* < 0.05), we found that: (a) HSPC-derived myeloid progenitors obtained from mice exposed to 0.1 Gy, a total of 976 proteins were down-regulated, while only one protein up-regulated; (b) HSPC-derived myeloid progenitors obtained from mice exposed to 0.25 Gy, a total of 33 proteins were down-regulated while 14 proteins were upregulated; and (c) HSPC-derived myeloid progenitors obtained from mice exposed to 0.5 Gy, a total of 58 proteins were down-regulated while 54 proteins were up-regulated. Details of these proteins can be found in the Supplementary Data A. 

**Figure 2:**
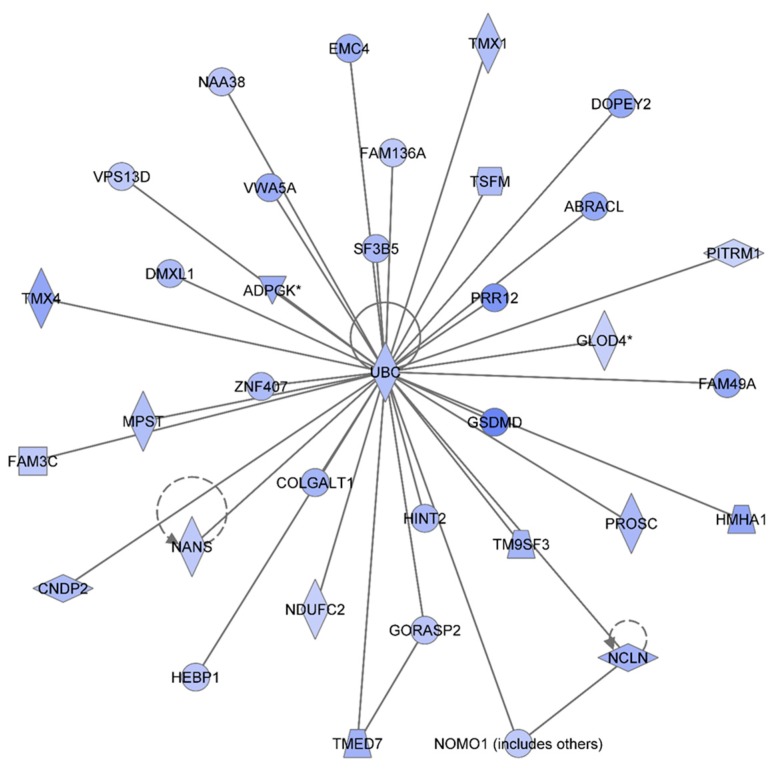
Ingenuity Pathway Analysis showing the top molecular Network in HSPC-derived myeloid cells collected at 1 week after exposure of mice to 0.1 Gy of ^48^Ti ions. This network is involved hereditary disorder, metabolic diseases, and neurological disease. It is constructed by 35 focus proteins: AABRACL (ABRA C-Terminal Like), ADPGK (ADP-Dependent Glucokinase), CNDP2 (CNDP Dipeptidase 2 (Metallopeptidase M20 Family), COLGALT1 (Collagen Beta1-*O*-Galactosyltransferase 1), DMXL1 (Dmx-Like 1), DOPEY2 (Dopey Family Member 2), EMC4 (ER Membrane Protein Complex Subunit 4), FAM136A (Family With Sequence Similarity 136, Member A), FAM3C (Family With Sequence Similarity 3, Member C), FAM49A (Family With Sequence Similarity 49, Member A), GLOD4 (Glyoxalase Domain Containing 4), GORASP2 (Golgi Reassembly Stacking Protein 2, 55kDa), GSDMD (Gasdermin D), HEBP1 (Heme Binding Protein 1), HINT2 (Histidine Triad Nucleotide Binding Protein 2), HMHA1 (Histocompatibility- Minor-HA-1), MPST (Mercaptopyruvate Sulfurtransferase), NAA38 (*N*-Alpha-Acetyltransferase 38, NatC Auxiliary Subunit), NANS (*N*-Acetylneuraminic Acid Synthase), NCLN (Nicalin), NDUFC2 (NADH Dehydrogenase (Ubiquinone) 1, Subcomplex Unknown 2, 14.5kDa), NOMO1 (NODAL Modulator 1), PITRM1 (Pitrilysin Metallopeptidase 1), PROSC (Proline Synthetase Co-Transcribed Homolog Bacterial), PRR12 (Proline Rich 12), F3B5 (Splicing Factor 3b, Subunit 5, 10kDa), TM9SF3 (Transmembrane 9 Superfamily Member 3), TMED7 (Transmembrane Emp24 Protein Transport Domain Containing 7), TMX1 (Thioredoxin-related transmembrane protein 1), TMX4 (Thioredoxin-related transmembrane protein 4), TSFM (Ts Translation Elongation Factor, Mitochondrial), UBC (Ubiquitin C), VPS13D (Vacuolar Protein Sorting 13 Homolog D), VWA5A (Von Willebrand Factor A Domain Containing 5A), and ZNF407 (Zinc Finger Protein 407). All of the focus proteins are down-regulated (blue IPA symbols).

**Figure 3:**
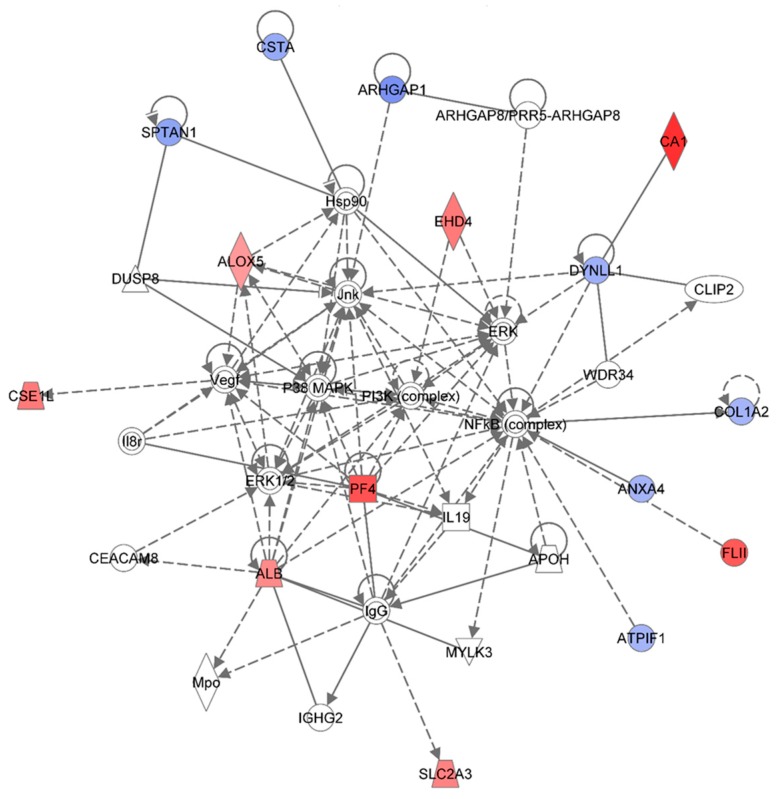
Ingenuity Pathway Analysis showing the top molecular network in HSPC-derived myeloid cells collected at 1 week after exposure of mice to 0.25 Gy of ^48^Ti ions. This network is involved lipid metabolism, small molecule biochemistry, and connective tissue disorders and developmental disorder. This network is constructed with 35 proteins: **ALB** (Albumin), **ALOX5** (Arachidonate 5-Lipoxygenase), **ANXA4** (Annexin A4), APOH (Apolipoprotein H-Beta-2-Glycoprotein I), **ARHGAP1** (Rho GTPase Activating Protein 1), **ARHGAP8** (Rho GTPase Activating Protein 8), **PRR5-ARHGAP8** (PRR5-ARHGAP8 Readthrough), ATPIF1 (ATPase Inhibitory Factor 1), **CA1** (Carbonic Anhydrase I), CEACAM8 (Carcinoembryonic Antigen-Related Cell Adhesion Molecule 8), CLIP2 (CAP-GLY Domain Containing Linker Protein 2), **COL1A2** (Collagen, Type I, Alpha 2), **CSE1L** (CSE1 Chromosome Segregation 1-Like), **CSTA** (Cystatin A or known as Stefin A), **DUSP8** (Dual Specificity Phosphatase 8), **DYNLL1** (Dynein, Light Chain, LC8-Type 1), **EHD4** (EH-Domain Containing 4), FLII (Flightless I Homolog), Hsp90 (Heat Shock Protein 90), IgG (Immunoglobulin G), IGHG2 (Immunoglobulin Heavy Constant Gamma 2-G2m Marker), IL19 (Interleukin 19), Il8r (Interleukin 8 Receptor beta), Jnk (c-Jun N-terminal protein kinases), MPO (Myeloperoxidase),MYLK3 (Myosin Light Chain Kinase 3), NF-κB (Nuclear Factor kappa B), MAPK (Mitogen-activated protein kinases), **PF4** (Platelet Factor 4), PI3K (Phosphoinositide-3-kinase), **SLC2A3** (Solute Carrier Family 2 (Facilitated Glucose Transporter), Member 3), **SPTAN1** (Spectrin, Alpha, Non-Erythrocytic 1), VEGF (Vascular endothelial growth factor), WDR34 (WD Repeat Domain 34). Among these, there are only 15 focus proteins (those in bold and shown in filled IPA symbols in the figure). Seven of the 15 focus proteins are down-regulated (those shown in blue IPA symbols), while eight of the 15 focus proteins are up-regulated (those shown in red IPA symbols).

**Figure 4:**
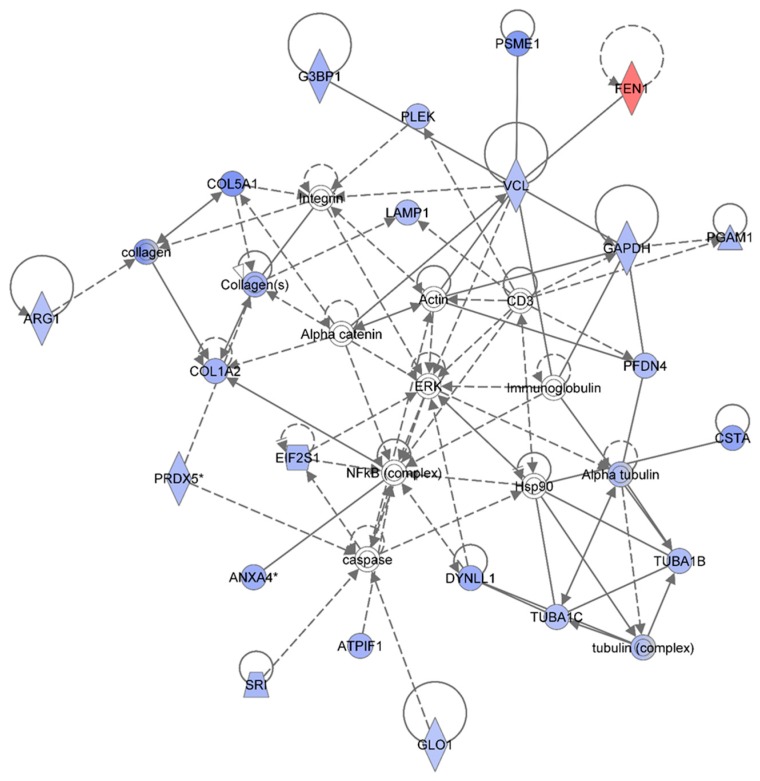
Ingenuity Pathway Analysis showing the top molecular network in HSPC-derived myeloid cells collected at 1 week after exposure of mice to 0.25 Gy of ^48^Ti ions. This network is involved in dermatological diseases and conditions, connective tissue disorders, and development disorder. It consists of 35 proteins: Actin, Alpha catenin, Alpha tubulin, **ANXA4** (Annexin A4), **ARG1** (Arginase 1), **ATPIF1** (ATPase Inhibitory Factor 1), **COL1A2** (Collagen, Type I, Alpha 2), **COL5A1** (Collagen, Type V, Alpha 1), **CSTA** (Cystatin A , also known as Stefin A), **DYNLL1** (Dynein, Light Chain, LC8-Type 1, **EIF2S1** (Eukaryotic Translation Initiation Factor 2, Subunit 1 Alpha, 35kDa), **FEN1** (Flap Structure-Specific Endonuclease 1), G3BP1 (GTPase Activating Protein (SH3 Domain) Binding Protein 1), **GAPDH** (Glyceraldehyde-3-Phosphate Dehydrogenase), **GLO1** (Glyoxalase I), **LAMP1** (Lysosomal-Associated Membrane Protein 1), **PFDN4** (Prefoldin Subunit 4), **PGAM1** (Phosphoglycerate Mutase 1), **PLEK** (Pleckstrin), **PRDX5** (Peroxiredoxin 5), **PSME1** (Proteasome (Prosome, Macropain) Activator Subunit 1-PA28 Alpha), SRI (Sorcin), **TUBA1B** (Tubulin, Alpha 1b), **TUBA1C** (Tubulin, Alpha 1c), **tubulin complex**, and **VCL** (Vinculin). Among these, there are only 21 focus proteins (those in bold and shown in filled IPA symbols shown in the figure). Twenty out of 21 focus proteins are down-regulated (those in blue IPA symbols). Only one focus protein is up-regulated (shown in red IPA symbol).

Table 1 summarizes the Ingenuity Pathway Analyses (IPA) analyses of identified proteins in each treatment group, as compared to the non-irradiated sham control group. These are top diseases and disorders, top molecular and cellular functions, top physiological system development and function, and top network. The results indicated that proteins involved in different diseases and disorders were found in each treatment group. The IPA analyses also presented various proteins with different molecular and cellular functions. Moreover, the IPA analyses provided information on the top networks involved in responses to 1 GeV/n ^48^Ti ions. Although more than one network was found for each dose of 1 GeV/n at one week post-exposure, we are presenting only the network with the highest score for each dose of 1 GeV/n ^48^Ti ions (as shown in Table 1). Figure 2, Figure 3 and Figure 4 show the top molecular networks detected by the IPA analyses in HSPC-derived myeloid cells after exposure of mice to 0.1, or 0.25, or 0.5Gy of 1 GeV/n ^48^Ti ions, respectively. The description of each IPA symbol has been previously described [35].

In each figure, only proteins that are considered to be focus proteins (those shown in filled symbols) will be discussed. The focus proteins are from the uploaded list and are available for generating networks. Other proteins appearing in the network are added by the IPA analysis software because the IPA database indicates that they interact with the focus proteins.

**Table 1:** The summary obtained from IPA analyses of identified proteins in HSPC-derived myeloid cells collected at one week after exposure of mice to various doses of 1 GeV/n ^48^Ti ions.

Radiation Dose (Gy)	Top Diseases and Disorders	Top Molecular and Cellular Functions	Top Physiological System Development and Function	Top Network
0.1	Infectious disease	Cell death and survival	Organismal survival	Hereditary Disorder, Metabolic Disease, Neurological Disease (score = 53)
	Neurological disease	RNA post-transcriptional modification	Immune cell trafficking	
	Psychological disorders	Cellular growth and proliferation	Hematological system development and function	
	Skeletal and muscular disorders	Protein Synthesis	Connective tissue development and function	
	Cancer	Nucleic acid metabolism	Tissue development	
0.25	Connective tissue disorders	Lipid metabolism	Hematological system development and function	Lipid metabolism, small molecule biochemistry, connective tissue disorders (score = 36)
	Dermatological diseases and conditions	Small molecule biochemistry	Immune cell trafficking	
	Developmental disorders	Cell death and survival	Skeletal and muscular system	
	Hereditary disorder	Cellular movement	Tissue development	
	Metabolic diseases	Amino acid metabolism	Digestive system development and function	
0.5	Cancer	Post-translational modification	Organismal development development and function	Dermatological diseases and conditions, connective tissue disorders, developmental disorder (score = 52)
	Hematological disease	Protein folding	Cardiovascular system	
	Immunological disease	Cell morphology	Connective tissue development and function	
	Dermatological diseases and conditions	Cellular assembly and organization	Embryonic development	
	Connective tissue disorders	Cell death and survival	Cell death and survival	

Figure 2 shows the IPA-analysis top network in HSPC-derived myeloid colonies collected at 1 week post-irradiation in response to 0.1 Gy of ^48^Ti ions. This network is related to heredity disorder, metabolic diseases, and neurological disease (score = 53). The solid lines represent the direct molecular interaction among proteins in the network. There are 35 focus proteins (those proteins that are from uploaded list and pass filters are applied). All of these proteins are shown in the filled IPA symbols. These proteins are associated with cell death and survival, RNA post-transcriptional modification, cellular growth and proliferation, protein synthesis, and nucleic acid metabolism). All of these 35 focus proteins shown in the network were down regulated and connected to ubiquitin C (UBC). Hence, the decreased levels of these proteins are likely to be due to ubiquination, the process known to be associated with protein degradation [36]. Of note, it is known that ubiquitination is important for several biological processes. These include: DNA repair, cell cycle, cell survival, differentiation, kinase modification, endocytosis, and regulation of other cell signaling pathways [37].

Figure 3 shows the IPA-analysis top network (score = 36) in HSPC-derived myeloid colonies collected at 1 week post-irradiation in response to 0.25 Gy of 1 GeV/n ^48^Ti ions. This network is involved lipid metabolism, small molecule biochemistry, and connective tissue disorders. The solid and dotted lines represent the direct and indirect molecular interaction among proteins in the network, respectively. Although there are 35 proteins in the network (Figure 3), only 15 proteins (those in filled IPA symbols) are uploaded from the list (focus proteins). Eight of these 15 proteins (shown in red color symbols) showed higher levels of expression than the control level. These proteins are albumim (ALB), arachidonate 5-lipoxygenase (ALOX5), carbonic anhydrase 1 (CA1), cellular apoptosis susceptible 1 (CSE1), EH domain-containing protein 4 (EHD4), flightless-II (FLII), platelet factor 4 (PF4), and solute carrier family 2 member 3 (facilitated glucose transporter 3) (SLC2A3).

Highly expression of ALB and CA were previously reported in BM cells of CBA/Ca or C57BL/6 mice exposed to γ-rays [15]. Our study is the first to report a high level of expression of ALOX5 protein in HSPC-derived myeloid colonies obtained from mice exposed to space radiation, although expression of ALOX5 is known to be critical regulator of chronic ML stem cells, both in humans and mice [38]. Taken together, these findings suggest that expression of ALOX5 after irradiation may play an important role in radiation-induced late health effects. Figure 3 also shows that an increased expression level of ALOX5 has a positive impact on the expression of heat shock protein 90 (Hsp90). Highly expressed SLC2A3 has been detected in several types of solid tumors such as colon [39] and brain [40] cancers, although its role in hematopoietic neoplasms has not been reported. It should be noted that these up-regulated proteins are associated, either directly or indirectly, to the nuclear factor-kappa B (NF-κB) complex, an important transcription factor involved in cell survival [41] and cell death [42] after exposure to radiation, depending on certain circumstances and cell type. Kinase activities from several sources facilitate such interaction: P38-mitogen-activated protein kinases (p38-MAPK), or phosphoinositide-3 (PI-3) kinase, or c-Jun N-terminal kinase (Jnk), or extracellular signal-regulated protein kinase (ERK). Figure 3 also shows seven focused proteins that their levels of expression were down-regulated, in relation with the control level 9Those in blue color). These proteins are: Annexin A4 (ANXA4), RHO GTPase-activator protein 1 (ARHGP1 or RHO 1), ATPase inhibitory factor 1 (ATPIF1), collagen, Type I, Alpha 2 (COL1A2), cystatin A (CSTA), dynein light chain 1 (DYNLL1), and spectrin (SPTAN1). The majority of down-regulated proteins are involved in cell death, cell survival, and cell proliferation (e.g., ANXA4, COL1A2, and CSTA). The results from IPA analysis also show that NF-κB, Jnk, ERK, and Hsp90 are involved in the down-regulation of these proteins.

Figure 4 shows the IPA-analysis top network (score = 52) in HSPC-derived myeloid colonies collected at 1 wk post-exposure in response to 0.5 Gy of 1 GeV/n ^48^Ti ions. The IPA analysis indicated that proteins in this network are involved dermatological diseases and conditions, connective tissue disorders, developmental disorder. Similar to Figure 3, the solid and dotted lines represent the direct and indirect molecular interaction among proteins in the network, respectively. Although there are 35 proteins shown in the network, only 21 proteins were the focus of this network (those in filled IPA symbols). Twenty of the focused proteins were down-regulated (those in blue symbols). Among these, five proteins associated with cell growth, cell survival and cell proliferation (*i.e.*, ANXA4, ATPIF1, COL1A2, CSTA, and DYNLL1) were also down-regulated in the 0.25 Gy-exposed mice previously shown in Figure 3. Several of the other down-regulated proteins were linked to actin, connective tissue (collagens) and cytoskeleton (tubulin) complexes.

Proteins that are linked to actin are: Glyceraldehyde 3-phosphate dehydrogenase (GAPDH), GTPase activating protein SH3 domain (G3BP1), phosphoglycerate mutase 1 (PGAM1), prefoldin (PFDN4), pleckstrin (PLEK), proteasome activator complex subunit 1 (PMSE1), and vinculin (VCL). These actin-associated proteins are also associated with embryonic development. The IPA results display a direct link among VCL, alpha-catenin, and actin that regulates cell-cell adhesion to ensure the stabilization of cell structure for synchronization of normal development. Hence, a reduction in these proteins after irradiation would disturb the homeostasis of the function of cells. The IPA results also show an association of VCLwith alpha-catenin, actin, and the collagen compartment (connective tissue) of the network in which several proteins are involved. Proteins involved in the collagen compartment are: Arginase (ARG1), COL1A2, collagen Type V Alpha 1 (COL5A1), lysosomal-associated membrane protein 1 (LAMP1, a protein regulates translation process), and peroxiredoxin (PRDX5). Normally, PRDX5 protein (an antioxidant enzyme) protects cells from radiation-induced death by suppression of redox-sensitive caspase activation [43]. Hence, a reduction in the level of PRDX5 in HSPC-derived myeloid colonies after exposure of mice to ^48^Ti ions is indicative of a higher level of cell death (via the caspase pathway). It is known that caspases are important for maintaining homeostasis through regulating cell death and inflammation [44]. Hence, dysregulation of caspases would trigger the induction of diseases such as cancer and inflammatory disorders.

The IPA analysis also reveals the down-regulation of sorcin (SRI, a calcium-binding protein) and glyoxalase 1-defense-enzyme (GLO1). These two proteins are also involved in defense mechanisms or detoxification through suppression of caspase activity. Overall, the results indicate that exposure to ^48^Ti ions diminishes the expression of several proteins involved in cell death and survival, mediated through suppression of caspase activity. It should be noted that caspase is linked to Hsp90, which interacts with the tubulin family (the cytoskeleton protein) that are involved in maintaining intracellular organization and normal cell movement. The IPA analysis shows that expression levels of four types of tubulins in HSPC-derived myeloid cells were reduced after exposure of mice to ^48^Ti ions. These proteins are: Alpha tubulin which directly binds to cysteine protease inhibitor (CSTA), tubulin alpha 1 C (TUBA1C), tubulin alpha 1B (TUBA1B), and tubulin complex (a heterodimer of tubulins alpha and beta that constitutes the promoter for microtubule assembly). Importantly, it should be noted that these down-regulated proteins are associated (either directly or indirectly) with the NF-κB complex and ERK, indicating the importance of these two signaling pathways after exposure to radiation.

One striking finding is that only one protein is up-regulated in this network, *i.e.*, flap structure specific endonuclease 1 (FEN1), shown in the red symbol. This protein is an enzyme involved in the base excision repair system, and its abnormality has been shown to be associated with tumor progression of mouse models with microsatellite instability [39]. Of note, FEN1 directly binds to VCL, an actin-binding, for its endonuclease activity. As mentioned previously, the level of VCL is decreased in HSPC-derived myeloid cells collected at 1 week after exposure of mice to ^48^Ti ions. Hence, it is possible that although the level of expression of FEN1 is high, its activity is defective since its protein partner, *i.e.*, VCL, is unavailable. This would result in an insufficient DNA repair process that, in turn, induces higher levels of damage.

### 3.2. Effects of 1 GeV/n ^48^Ti Ions on HSPC-Derived Myeloid Colonies Collected at 6 Months Post-irradiation

For all exposed groups, a total of 1988 unique and non-redundant proteins were identified with ≥90% confidence (Supplemental data A). Among the 1988 identified, a total of 578 proteins were differentially expressed based on the one-way analysis of variance (ANOVA, *p* < 0.01). For the multiple comparisons among groups (the Holm-Sidak method, with significant differences at *p* < 0.05), we found that: (a) HSPC-derived myeloid progenitors obtained from mice exposed to 0.1 Gy, a total of 12 proteins are down-regulated, while no up-regulated protein; (b) HSPC-derived myeloid progenitors obtained from mice exposed to 0.25 Gy, three proteins are down-regulated while 10 proteins are up-regulated; and (c) HSPC-derived myeloid progenitors obtained from mice exposed to 0.5 Gy, a total of two proteins were down-regulated while 80 proteins are up-regulated. Details of up- and down-regulated proteins found in each treatment group can be found in the Supplemental Data B.

**Figure 5:**
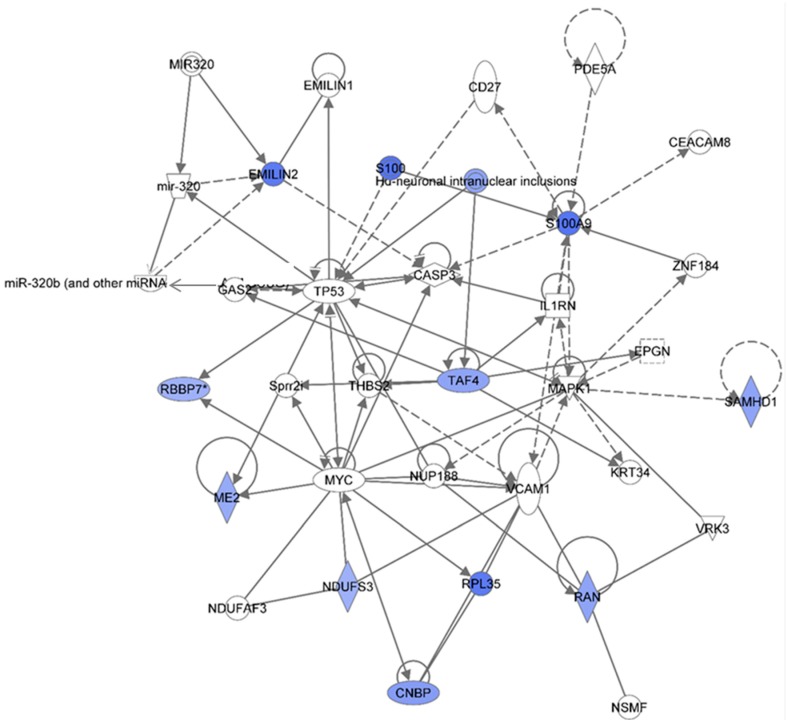
Ingenuity Pathway Analysis showing the top molecular Network in HSPC-derived myeloid cells collected at 6 mos after exposure of mice to 0.1 Gy of ^48^Ti ions. This network is involved cancer, tumor morphology, cardiac necrosis/cell death (score = 29). It is constructed by 35 proteins: CASP3 (Caspase 3, Apoptosis-Related Cysteine Peptidase), CD27 (CD27 Molecule), CEACAM8 (Carcinoembryonic Antigen-Related Cell Adhesion Molecule 8), **CNBP** (CCHC-Type Zinc Finger, Nucleic Acid Binding Protein), EMILIN1 (Elastin Microfibril Interfacer 1), **EMILIN2** (Elastin Microfibril Interfacer 2), EPGN (Epithelial Mitogen), GAS2 (Growth Arrest-Specific 2), **Hu (histone-like protein protein)-neuronal intranuclear inclusions**, IL1RN (Interleukin 1 Receptor Antagonist), KRT34 (Keratin 34, Type I), MAPK1 (Mitogen-Activated Protein Kinase 1), **ME2** (Malic Enzyme 2, NAD(+)-Dependent, Mitochondrial), miR320 (microRNA 320) and others, MYC (V-Myc Avian Myelocytomatosis Viral Oncogene Homolog), NDUFAF3 (NADH Dehydrogenase Ubiquinone Complex I, Assembly Factor 3), **NDUFS3** (NADH Dehydrogenase Ubiquinone Fe-S Protein 3, 30kDa NADH-Coenzyme Q Reductase), NSMF (NMDA Receptor Synaptonuclear Signaling And Neuronal Migration Factor), NUP188 (Nucleoporin 188 kDa), PDE5A (Phosphodiesterase 5A, CGMP-Specific), **RAN** (ras-related nuclear protein), **RBBP7** (Retinoblastoma Binding Protein 7), **RPL35** (Ribosomal Protein L35), **S100** (S100 Calcium Binding Protein), **S100A9** (S100 Calcium Binding Protein A9**)**, **SAMHD1** (SAM Domain And HD Domain 1), Sprr2i (Small proline-rich protein 2I), **TAF4** (TAF4 RNA Polymerase II, TATA Box Binding Protein (TBP)-Associated Factor, 135kDa), THBS2 (Thrombospondin 2), TP53 (Tumor Suppression Protein P53), VCAM1 (Vascular Cell Adhesion Molecule 1), VRK3 (Vaccinia Related Kinase 3), and ZNF184 (Zinc Finger Protein 184). Among these proteins, there are only 12 focus proteins (those in bold and shown in the filled IPA symbols in the figure). All of the focus proteins are down-regulated (IPA symbols filled with blue color).

**Figure 6:**
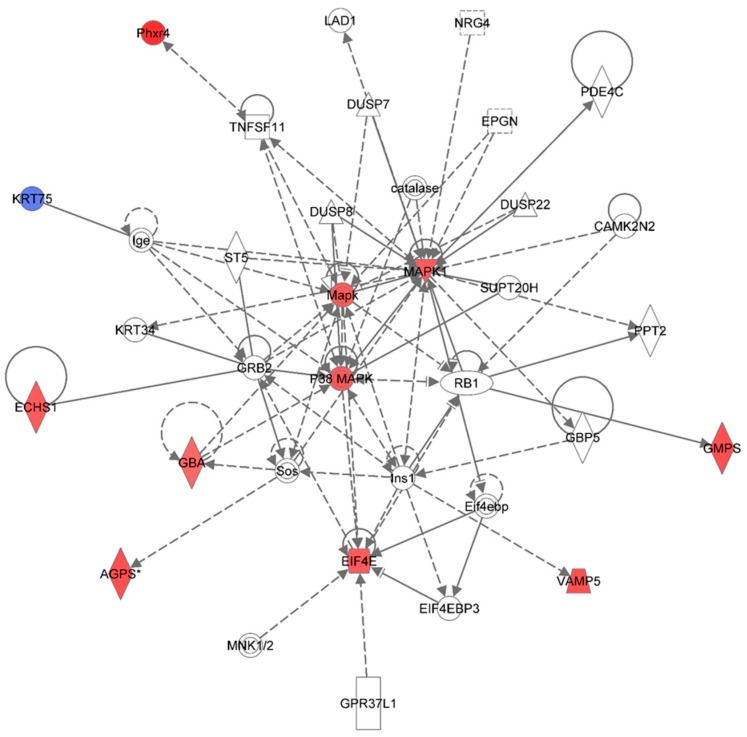
Ingenuity Pathway Analysis showing the top molecular network in HSPC-derived myeloid cells collected at 6 months after exposure of mice to 0.25 Gy of ^48^Ti ions. This network is involved cell death and survival, cell cycle, and connective tissue development/function. It is constructed with 35 proteins: **AGPS** (Alkylglycerone phosphate synthase), CAMK2N2 (Calcium/Calmodulin-Dependent Protein Kinase II Inhibitor 2), catalase (Catalase), DUSP7 (dual specificity phosphatase 7), DUSP8 (dual specificity phosphatase 8), DUSP22 (dual specificity phosphatase 22), **ECHS1** (Enoyl CoA hydratase, short chain, 1, mitochondrial), **EIF4E** (Eukaryotic translation initiation factor 4E), EIF4EBP3 (Eukaryotic Translation Initiation Factor 4E Binding Protein 3), EPGN (Epithelial Mitogen Homologue), **GBA** (Glucosidase, beta, acid), GBP5 (Guanine Nucleotide-Binding Protein 5), **GMPS** (Guanine monphosphate synthase), GPR37L1 (G Protein-Coupled Receptor 37 Like 1), GRB2 (Growth factor receptor-bound protein 2), Ige **(**Immunoglobulin E**)**, Ins1 (Insulin 1), KRT34, **KRT75** (keratin 75), **MAPK** or **Mapk** (Mitogen-activated protein kinase), **p38 MAPK** (p38 MAPK-activated protein kinases), **MAPK1** (mitogen-activated protein kinase 1), PDE4C (Phosphodiesterase 4C, cAMP-specific), **Phxr4** (Per-hexamer repeat gene 4), PPT2 (Palmitoyl-Protein Thioesterase 2), RB1 (retinoblastoma 1), Sos (Son of Sevenless-encoding guanine nucleotide exchange factors, ST5 (Suppression of tumorigenicity 5), SUPT20H (Transcription factor SPT20 homolog), TNFSF11 (Tumor Necrosis Factor (Ligand) Superfamily, Member 11), and **VAMP5** (vesicle-associated membrane protein 5**)**. Among these proteins, there are only 11 focus proteins (those in bold and shown in the filled IPA symbols in the Figure). Ten out of 11 focus proteins are down-regulated (shown blue IPA symbols). Only one focus protein is up-regulated (shown in red IPA symbols).

**Figure 7:**
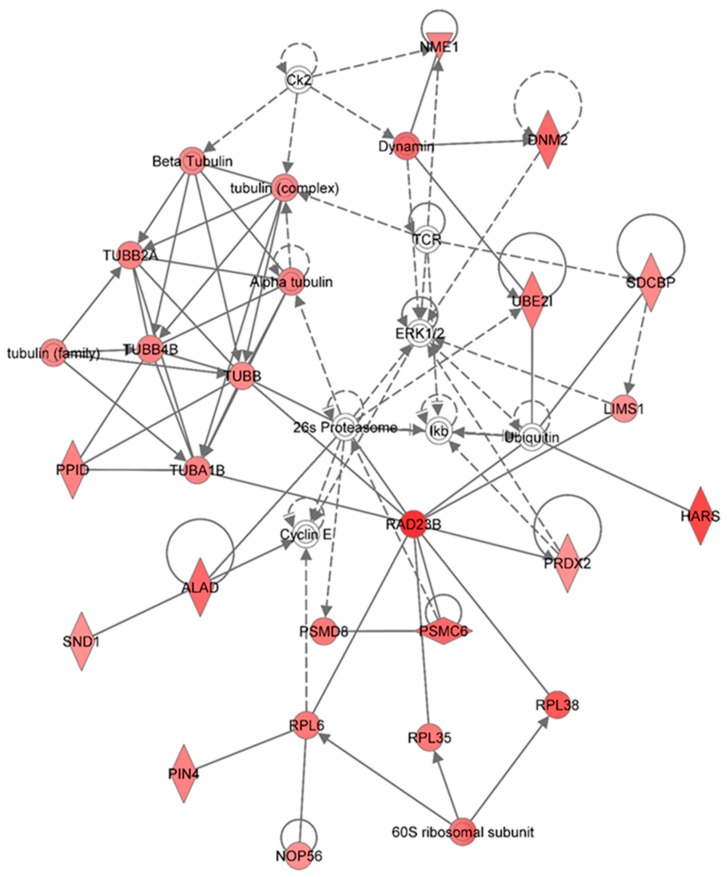
Ingenuity Pathway Analysis showing the top molecular network in HSPC-derived myeloid cells collected at 6 months after exposure of mice to 0.5 Gy of ^48^Ti ions. This network is involved cancer, hematological disease, and immunological diseases. It is constructed with 35 proteins: 26s Proteasome, **60S ribosomal subunit**, **ALAD** (Aminolevulinate dehydratase), **Alpha tubulin**, **Beta Tubulin**, Ck2 (Casein Kinase 2), Cyclin E, **DNM2 (**Dynamin 2**)**, **Dynamin**, ERK1/2 (Extracellular-signal-regulated kinases 1/2), **HARS** (Histidyl-tRNA synthetase), Ikb (Nuclear factor of kappa light polypeptide gene enhancer in B-cells inhibitor), **LIMS1** ((LIN-11, ISL-1, MEC-3 and Senescent Cell Antigen-Like Domains 1), **NME1** (Non-Metastatic Cells 1), **NOP56** (Nucleolar protein 56), **PIN4** (Peptidylprolyl Cis/Trans Isomerase, NIMA-Interacting 4), **PPID** (peptidylprolyl isomerase), **PRDX2** (Peroxiredoxin 2), **PSMC6** (Proprotein Convertase Subtilisin/Kexin Type 6), **PSMD8** (Proteasome or Prosome or Macropain, Subunit, Beta Type 8), **RAD23B** (RAD23 Homolog B), **RPL6** (Ribosomal Protein L 6), **RPL35** (Ribosomal Protein L 35**)** , **RPL38** (Ribosomal Protein L 38)**, SDCBP** (Syndecan Binding Protein or Syntenin), **SND1** (Staphylococcal Nuclease And Tudor Domain Containing 1), TCR (T cell receptor), TUBA1B (Tubulin, Alpha 1b), **TUBB** (Tubulin, Beta), **TUBB2A (**Tubulin, Beta 2A Class IIa), **TUBB4B (**Tubulin, Beta 4A Class IV b), **tubulin (complex)**, **tubulin (family)**, **UBE2I** (Ubiquitin-Conjugating Enzyme E2I), and Ubiquitin. Among these proteins, there are only 28 focus proteins (those in bold and shown in the filled IPA symbols in the Figure). All of the focus proteins are up-regulated (IPA symbols filled with red color).

Table 2 summarizes the Ingenuity Pathway Analyses (IPA) of identified proteins in each treatment group, as compared to the non-irradiated sham control group. These are top diseases and disorders, top molecular and cellular functions, top physiological system development and function, and top network. Similar to the data from the 1-week harvest time-point, the results indicate different proteins were found in each treatment group. The IPA analyses also presented proteins with different molecular and cellular functions. Moreover, the IPA analyses provided information on the top molecular networks involved in responses to different doses of 1 GeV/n ^48^Ti ions. Similar to the data for the 1-week harvest time-point, we are presenting only the molecular network with the highest score for each dose of 1 GeV/n ^48^Ti ions. Figure 5, Figure 6 and Figure 7 show the top molecular networks detected by the IPA analyses in HSPC-derived myeloid cells collected at 6 mos after exposure of mice to 0.1, or 0.25, or 0.5Gy of 1 GeV/n ^48^Ti ions, respectively. As previously mentioned, the description of each IPA symbol has been described [35]. 

**Table 2:** The summary obtained from IPA analyses of identified proteins in HSPC-derived myeloid cells collected at six months after exposure of mice to various doses of 1GeV/n ^48^Ti ions.

Radiation Dose (Gy)	Top Diseases and Disorders	Top Molecular and Cellular Functions	Top Physiological System Development and Function	Top Networks
0.1	Connective tissue disorders	Cellular movement	Connective tissue development and function	Cancer, tumor morphology, cardiac necrosis/cell death (score = 29)
	Dermatological diseases and conditions	Cellular growth and proliferation	Embryonic development	
	Hereditary disorder	Lipid metabolism	Hematological system development and function	
	Immunological disease	Small molecule biochemistry	Organismal development	
	Inflammatory disease	Cell to cell signaling and interaction	Tissue development	
0.25	Cancer	Carbohydrate metabolism	Cardiovascular system development and function	Cell death and survival, cell cycle, connective tissue development and function (score = 25)
	Connective tissue disorders	Cell cycle	Hair and skin development and function	
	Dermatological diseases and conditions	Cell death and survival	Hepatic system development and function	
	Developmental disorders	Cell morphology	Tissue development	
	Hereditary disorder	Cell-to-cell signaling and interaction	Tissue morphology	
0.5	Cancer	Ribonucleic acid (RNA) post-transcriptional modification	Digestive system development and function	Cancer, hematological disease, immunological disease (score = 48)
	Hematological disease	Cellular development	Embryonic development	
	Immunological disease	Cellular growth and proliferation	Hair and skin development and function	
	Organismal injury and abnormalities	Cell death and survival	Hematological system development and function	
	Respiratory disease	Gene expression	Hematopoiesis	

The description of the focus proteins has previously been presented in samples collected at 1 week post-irradiation. Similarly, only proteins that are considered to be the focus protein by the IPA analysis (those in filled IPA symbols) will be discussed.

Figure 5 shows the IPA-analysis of identified proteins HSPC-derived myeloid colonies collected at 6 months post-irradiation in response to 0.1 Gy of ^48^Ti ions. The results show that this network is involved in cancer, tumor morphology, and cardiac necrosis/cell death (score = 29). The solid and dotted lines represent the direct and indirect molecular interaction among proteins in the network, respectively. Although this network is constructed with 34 proteins, there are only 12 focus proteins (those in filled IPA symbols). All of the focus proteins are down-regulated (blue symbols). The majority of these proteins are enzymes and transcription regulators.

Down-regulation of elastin microfibril inter-located protein 2 (EMILIN2) was detected in HSPC-derived myeloid cells obtained at 6 months after exposure of CBA/CaJ mice to 1GeV/n ^48^Ti ions. EMILIN2 is an extracellular matrix (ECM) glycoprotein in the EMILIN family. Of note, tumor suppressor activity of EMILIN has been suggested [45]. Expression of EMILIN2 has been detected in several tissues of mice (*i.e.*, lung, heart, aorta and bone marrow), with the highest expression in bone marrow. It is also known that EMILIN2 is a binding partner of EMILIN1 [46]. Recently, it has been found that EMILIN2 regulates platelet activation, thrombus formation and clot retraction [47]. Hence, deficiency or reduction in EMILIN2 would impair platelet aggregation response. Of note, the IPA analyses indicate that TP53 and microRNA320 and microRNA320b directly regulate the expression of proteins in the EMILIN family.

The IPA analysis also shows that the expression of S100 and S100A9 are down-regulated. The S100 proteins (a multigenic-family of Ca^2+^ binding proteins, e.g., S100A1, S100A2, S100A8, and S100A9) are involved in several intracellular and extracellular regulatory activities, e.g., enzyme activities, dynamics of cytoskeleton constituents, cell growth and differentiation, cell cycle, and Ca^2+^ homeostasis [48]. It has been reported that some proteins in the S100 family (*i.e.*, S100A1, and S100A2) bind to TP53 proteins for proper transcriptional activity of tumor suppressor protein 53 (TP53) [49]. Hence, down-regulation of the S100 proteins would affect the normal activity of TP53. The S00A9 (also known as MRP14, migration inhibitory factor-related protein 14) is tightly regulated during differentiation of myeloid cells and is essential for myeloid cell function [50]. Hence, a reduction in S100A9 after exposure to 1 GeV/n ^48^Ti ions suggests dysregulation of the function of myeloid cells such as cytokines production in response inflammatory stimulation. It is shown in the network that the activity of S100A9 is dependent to MAPK and zinc finger proteins 184 (ZNF184).

Low levels of cellular nucleic acid protein (CNBP) may reduce rate of global protein synthesis, thereby reducing proliferation and increasing apoptosis. Further, the expression level of Ras-related nuclear protein (RAN), a nuclear transport protein, is also down-regulated, that may reflect a reduced cell proliferation. Down-regulation of a mitochondrial NAD-dependent maleic enzyme 2 (ME2) is detected. An increased in the level of reactive oxygen species (ROS) has been found to be linked to a reduction in the level of ME2 [51]. Hence, down-regulated ME2 may reflects the persistent increases in the level of ROS in HSPC-derived myeloid cells collected at 6 mos after exposure of mice to 0.1 Gy of 1 GeV/n ^48^Ti ions that, in turn, lead to late-occurring damage such as chromosome aberrations or aberrant pattern of DNA methylation [52]. Further, similar to the HSPC-derived myeloid cells collected at 1 wk post-irradiation), down-regulation of proteins in the family of NADH dehydrogenase (ubiquinone) Fe-S protein (NDUFS), a protein involved in ROS regulation [53] is found. The IPA analyses reveal that expression of ME2, NDUFS3, RAN, retinoblastoma binding protein 7 (RBBP7, involved in cell proliferation and differentiation), and ribosomal protein L35 (RPL35) are modulated by the MYC protein. Of note, expression of the RBBP7 protein is directly regulated by TP53 protein.

Down-regulation of the SAM domain-and HD domain-containing protein 1 (SAMHD1) was detected. The SAMHD1 has deoxynucleoside triphosphate triphosphohydrolase and 3′→5′ exonuclease activity. It is known that SAMHD1 protects host cells from viral infection and DNA damage. It also has been reported that deficiency in SAMHD1 expression results in a complex inherited autoimmune inflammatory encephalopathy disease namely Aicardi-Goutières syndrome [54]. Further, it is known that SAMHD1 a major regulator of DNA precursor pools in mammalian cells [55]. Recently, it has been found that SAMHD1 plays a role in DNA repair [56]. Taken together, the reduction in expression level of SAMHD1 would interfere with normal homeostasis of the cells. The IPA analysis indicates that MAPK affects the normal function of SAMHD1. Of note, the IPA analysis also indicates a reduction in the level of Hu-neuronal intranuclear inclusions in HSPC-derived myeloid cells after exposure of CBA/Ca mice to 0.1 Gy of 1GeV/n ^48^Ti ions. This protein directly binds to transcription initiation factor TFIID subunit 4 (TAF4), a protein involved in transcription initiation. The role of these two proteins in response to ^48^Ti-ion -exposure is unclear.

Figure 6 shows the top network resulting from the IPA-analysis of identified proteins in HSPC-derived myeloid cells collected at 6 months post-irradiation in response to 0.25 Gy of ^48^Ti ions. As shown in Table 2, this network is involved cell death and survival, cell cycle, connective tissue development and function (score = 25). The solid and dotted lines represent the direct and indirect molecular interactions among proteins in the network, respectively. Although this network is constructed with 35 proteins, there are only 11 focus proteins (those in filled IPA symbols). Ten focus proteins are up-regulated (those shown in symbols filled with red color). Only one focus protein (keratin 75 or KRT75), which is shown in the symbol filled with blue color, is down-regulated. It is known that keratin is expressed in hair follicles and that forms major cytoskeleton in all embryonic and adult epithelia [57]. However, increasing evidence suggests that keratins can act as scaffolds to regulate cell growth and survival in epithelial cells. It has been found that KRT75 is involved in the differentiation of hematopoietic-progenitor cells through a combination of mechanical and signaling mechanisms [58]. Hence, it is possible that down-regulation of KT75 may be attributed to the abnormal differentiation process of hematopoietic-progenitor cells.

In this network, the MAPK is the major kinase signaling pathway involved in up-regulation of several proteins with various functions such as enzymes and regulators of the translational process. The enzymes that are up-regulated are: Alkylglycerone phosphate synthase (AGPS), enoyl co-enzyme A hydratase (ECHST, a mitochondrial enzyme), glucosidase (GBA), and guanine monophosphate synthase (GMPS), and vesicle-associated membrane protein 5 (VAMP5) and putative per hexamer repeat protein 4 (Phxr4).

One of the important regulators of the translational process that is up-regulated in HSPC-myeloid cells by the MAPK signaling pathway after exposure of mice to 1 GeV/n 48Ti ions is eukaryotic translation initiation factor 4E (EIF4E, the rate-limiting component in protein synthesis). The EIF4E protein plays an important role in translational regulation in response to stress and apoptosis that may lead to cell/tissue damage [59]. Over-expression of EIF4E is linked to cancer progression and drives cellular transformation, tumorigenesis, and metastatic progression [60]. Hence, EIF4E is frequently overexpressed in several types of human cancers, both solid and hematopoietic neoplasms [60,61]. Such oncogenic potential of EIF4E arises from its ability to bind the 7-methyl guanosine (m7G) cap on mRNAs, thereby selectively enhancing EIF4E-dependent nuclear mRNA export and translation [62]. Hence, it is plausible to speculate that over-expression of EIF4E is associated with the induction of genomic instability after exposure to ^48^Ti ions. Since targeting the expression of EIF4E for cancer therapy is being actively investigated, it is possible to search for EIF4E inhibitors to serve as radiation countermeasures to protect cells/tissues from radiation-induced damage.

Figure 7 shows the top network resulting from the IPA-analysis of identified proteins in HSPC-derived myeloid cells collected at 6 months in response to 0.5 Gy of 1 GeV/n ^48^Ti ions. As shown in Table 2, the results show that this network is involved in cancer, hematological disease, and immunological disease (score = 48). As with other networks, the solid and dotted lines represent the direct and indirect molecular interactions among proteins in the network, respectively. This network is constructed with 35 proteins, but only 28 of these are focus proteins (shown in filled IPA symbols with red color). All of the focus proteins are up-regulated. The majority of proteins involved in this network are the tubulin family, proteasome/ubiquination members, kinases (ERK or I kappa B, normally known as IκB which is a regulator of NF-κB activation). Intriguingly, proteins in the tubulin family are down-regulated in HSPC-derived myeloid cells collected at an early time-point (1 week post-exposure, as shown previously in Figure 2) but they are up-regulated in these cells collected at 6 months post-irradiation, at which time genomic instability has been detected [52]. Hence, it is possible that up-regulation of tubulin proteins (which are the component of microtubules) has a role in the induction of genomic instability after exposure to 1 GeV/n ^48^Ti ions. This statement is supported by previous reports showing a link between deregulation of microtubules/tubulins (such as an increased microtubule assembly) and genomic instability [63] and cancer [64]. It should be noted that the proteasome/ubiquination pathway is the major pathway to regulate the level of tubulin proteins, which is also directly linked to the inhibitor-kappa B (IκB), a regulator of NF-κB activation.

Other proteins that directly interact with proteasome/ubiquinition pathways are RAD23B (a nucleotide excision repair enzyme) and several enzymes that are that are required for the maintenance of basic cellular function. These enzymes include: Peptidyl-prolyl *cis*–*trans* isomerase D (PPID, also known as Cyclophilin-type PPIase, known to accelerate protein folding), Peptidyl-prolyl *cis*–*trans* isomerase NIMA-interacting 4 (PIN4), and aminolevulinate dehydratase (ALAD, known to response to oxidative stress induced by toxix agents such as radiation or lead), staphylococcal nuclease domain-containing protein 1 (SND1, known to regulate gene expression and promote tumor angiogenesis. In addition to proteasome/ubiquitin pathway, the RAD23B protein also interacts directly with several molecules involved in protein synthesis, *i.e.*, 60S ribosomal subunit, ribosomal protein L (RPL) 6, 35, and 38, including nucleolar protein 56. As well as molecules involved in proteasome/ubiquitinin pathway and in protein synthesis, RAD23B interact with peroxiredoxin 2 (PRDX2, an antioxidant enzyme), which is also up-regulated. Similar to other anti-oxidant proteins (e.g., manganese superoxide dismutase, MnSOD [65]), a high level of PRDX2 has been found in breast cancer cells [66], conceivably due to the anti-apoptotic and enhance cell proliferation of these enzymes plausibly. Further, a high level of MnSOD also plays a role in radiation-induced genomic instability [67]. Hence, it is plausibly to speculate that a high level of PRDX2 expression detected in HSPC-derived myeloid cells has a role in the induction of genomic instability after exposure of mice to 1GeV/n ^48^Ti ions. Recently, highly expressed PRDX2 has been observed in neutrophils of myelodysplastic syndrome (MDS) patients with refractory cytopenia and multilineage dysplasia, in relation to that of healthy control subjects [68]. It is known that MDS patients are at a high risk for developing acute ML. Hence, it is reasonable to speculate that the expression level of PRDX2 protein would be high in acute ML patients. However, it has been reported that PRDX2 is suppressed in acute ML patients [69]. Of note, it is unknown if radiation is the causal factor for the induction of acute ML in these patients. Hence, these findings warrant further investigation of the role of PRDX2 in radiation-induced acute ML.

Other proteins that are up-regulated and interact with proteasome/ubiquitin and kinase pathways include: Proteins in the dynamin family (a GTPase enzyme responsible for endocytosis of cells), histidyl-tRNA synthetase (HARS), LIM and sebescent cell and antigen-like domain 1 (LIMS1), nucleoside diphosphate kinase A (NME1), and syntenin or syndecan binding protein (SDCBP).

## 4. Conclusions

This is the first study to investigate protein expression profile in the colonies of HSPC-derived myeloid progenitors (the best population of cells for studying *in vivo* biological effects of radiation on myeloid stem cells and the target cell for radiation-induced ML) after a whole body exposure of mice to 1 GeV/n ^48^Ti ions. We used the label-free quantitative mass spectrometry (LFQMS), proteomic approach developed in our laboratory, to identify proteins and to determine the alterations in their expression levels (up- or down-regulation) in HSPC-derived myeloid cells collected at an early time-point (1 week) and at a late time-point (6 months) post-exposure. Our LFQMS proteomic platform is an innovative, experimentally-based method that accurately determines peptide peak retention time and uses multiple filters for exclusion of unqualified peptides by peptide frequency, retention time, intensity coefficient of variation, and intensity correlation to enhance protein quantification of qualified peptides and proteins. Our data demonstrate that the patterns of protein expression profiles are dose and time dependent. The majority of proteins with altered expression levels are involved in cell cycle control, cellular growth and proliferation, cell death and survival, cell-to-cell signaling and interaction. The IPA analyses indicate several important processes involved in responses to exposure to ^48^Ti ions. These include the proteosme/ubiquination, protein synthesis, post-translation modification, and lipid metabolism. These processes affect the development and function of hematological system, immune cell trafficking, including the cytoskeleton. The majority of proteins included in the top networks detected in samples collected at late-time point (6 months post-irradiation) are associated with cancer, hematological diseases, and immunological diseases. Our data strongly indicate the involvement of the NF-κB and MAPKs (ERKs, JNKs, and p38MAPK) pathways in signal transduction in HSPC-derived myeloid cells after exposure to 1 GeV/n ^48^Ti ions. The findings from our study provide an important basis for future discovery of compounds that could potentially be highly valuable to counteract radiation-induced damage. For example, inhibitors of protein translation machinery (e.g., those specific for the inhibition of ELFI4) should be beneficial for such purpose since the translational process is an essential step in response to radiation exposure.

## Supplementary Materials

Supplementary File 1

Supplementary File 2
